# Copeptin as biomarker for acute ischemic stroke prognosis and revascularization treatment efficacy

**DOI:** 10.3389/fneur.2024.1447355

**Published:** 2024-12-13

**Authors:** Antonia Ioana Vasile, Cristina Tiu, Corin Badiu

**Affiliations:** ^1^Neurology Department, University Emergency Hospital of Bucharest, Bucharest, Romania; ^2^Carol Davila University of Medicine and Pharmacy, Bucharest, Romania; ^3^Endocrinology IV, C.I. Parhon National Institute of Endocrinology, Bucharest, Romania

**Keywords:** copeptin, acute ischemic stroke, stroke prognosis, stroke biomarkers, treatment efficiency in stroke patients

## Abstract

**Introduction:**

Pro-arginine vasopressin consists of three peptides: *arginine-vasopressin, neurophysin II, and copeptin*. AVP is released by the neurohypophysis in response to increased plasma osmolality, decreased blood volume and stress. Copeptin has the advantage of being stable ex vivo and easy to measure. New data show the importance of copeptin in ischemic stroke and its complications.

**Methods:**

We present a literature review that highlights the importance of copeptin as a biological marker for stroke. We searched the Pubmed and Scopus databases for papers with the following keywords: “stroke AND copeptin.” PRISMA criteria were used.

**Results:**

We identified 332 papers that met the criteria. We excluded analyzed reviews, systematic reviews and meta-analyses. 31 articles resulted. The number of patients included in the analyzed studies varied between 18 and 4,302. Copeptin is a marker that associated with clinical stroke severity, infarct volume, short-term and long-term functionality and mortality and adds prognostic value to the previously used scales. It may reflect the effectiveness of revascularization therapy. Copeptin is a biomarker that can help predict post-stroke complications such as: cerebral edema and hemorrhagic transformation.

**Discussion:**

Copeptin is a novel and promising biomarker for evaluating cerebrovascular diseases. Because it is considered a non-specific biomarker, it is not yet used routinely and it cannot replace the clinical examination. However, combined with other clinical or paraclinical parameters, it can increase the accuracy of the diagnosis.

## 1 Introduction

Stroke is globally recognized as an important cause for mortality and disability (1). Ischemic stroke is the second leading cause of death in elderly patients over 60 years old and the fifth leading cause of death in patients aged 15 to 59 years (2). It is the leading cause of long-term physical and cognitive disability (3).

Survivors often require long-term care and are at a higher risk of recurrent stroke, compared to normal controls (4). This is why there is a need for rapidly measurable biomarkers that can predict stroke prognosis in order to optimize care and allocation of healthcare resources.

Although much progress has been made in the clinical and imaging management of stroke patients, there is still a lack of biological markers that can be used in the diagnosis and prognosis of these patients. Blek et al. (5) mention several markers associated with the prognosis of these patients, among which are: markers of *inflammation* (procalcitonin and mannose binding lectin), markers of *atherogenesis* (adipocyte fatty acid-binding protein), markers that demonstrate *stress response* (copeptin and cortisol) and natriuretic peptide. Other biomarkers used to evaluate functional outcome such as: mid-regional pro-atrial natriuretic peptide (MRproANP), mid-regional proadrenomedullin (MRproADM), C-terminal proendothelin, interleukin-6 (IL-6), interleukin-8 (IL-8), interleukin-10 (IL-10), lipopolysaccharide-binding protein (LBP), human leukocyte antigen-DR isotype (HLA-DR) (1, 6), C-reactive protein (CRP) (7–9), matrix metalloproteinase-9 (MMP-9) (10), fibrinogen (FBG) (11) and brain natriuretic peptide (12).

Thrombolysis and mechanical thrombectomy are the only effective treatments for patients with acute ischemic stroke that arrive at the hospital in the early stages (13, 14). However, the efficacy of both treatments are time-dependent, so the fast diagnosis of ischemic stroke is required (15). Clinical diagnosis of stroke in the emergency room can be challenging and, most of the time, requires brain imaging. Computer tomography (CT) or magnetic resonance imaging (MRI) are the only paraclinical evaluations that could rule out hemorrhagic stroke or other pathologies that mimic stroke. But there are some situation (such as lack of radiology department availability) when the time-critical decision to start treatment is based only on the clinical presentation (15). In these situation, biomarkers that diagnose or differentiate stroke may be helpful. One of the biomarkers that gained attention recently is copeptin (5, 15–19).

Pro-arginine vasopressin (pro-AVP) is produced by the supraoptic and paraventricular nuclei in the hypothalamus and acts as a precursor for 3 peptides: *arginine-vasopressin, neurophysin II*, *and copeptin* (20). Arginine-vasopressin (AVP) or antidiuretic hormone (ADH) is released by the neurohypophysis in response to stress, increased plasma osmolality or decreased blood volume (21). While AVP receptor antagonists are studied for their cerebral protective effects, they are not typically administered as standard treatment in stroke but may hold promise in managing cerebral edema or related complications (22).

Copeptin can be identified in circulation in equimolar amounts with AVP (23–25). Measuring the blood concentration of AVP is complicated given that it is an *unstable* peptide (plasma half-life of 5–15 min) and is *bound to platelets* (26, 27). On the other hand, copeptin is a *stable* molecule and *easy to measure* (28). Copeptin may be a useful biomarker in the Emergency Room because the assay can be available within 60 min only (29). Median copeptin levels for healthy controls range from 3.7 to 4.2 pmol/L (24).

Botros et al. (30) evaluated copeptin levels at 64 patients who presented to the emergency room for acute illness such as: chronic liver disease, chronic obstructive pulmonary disease (COPD), stroke or decompensated heart failure in comparison to healthy controls. They reported that there was a significant difference between survivors and non-survivors of stroke patients. The researchers also demonstrated that a high copeptin level is associated to longer hospital stay and a poor outcome (30). Also, it was demonstrated that copeptin levels were lower in non-stressed healthy controls in comparison with hospitalized patients and with patients that went under significant surgical interventions associated with a high level of stress, this result reflecting that copeptin is influenced by the individual stress level (31).

Studies have shown that elevated copeptin levels correlate with prognosis and may help in the differential diagnosis prior to imaging in several cardiovascular pathologies such as: acute myocardial infarction, congestive heart failure, ischemic stroke, aneurysmal subarachnoidal hemorrhage and head trauma (32–36).

AVP has an important role in the development of cerebral edema (37), which increases the severity of a stroke (38). Kozniewska & Romaniuk (39) explained that vasopressin prevents brain cell adaptation to hyponatremia and participates in vasogenic edema and cellular ballooning after stroke. Vakili et al. (40) demonstrated that *vasopressin administration exacerbates acute ischemic cerebral edema*. Moreover, it was shown that *treatment with vasopressin receptor blocker drugs reduces cerebral edema* (41). Thus, it was postulated that *measuring copeptin can have prognostic significance in the development of cerebral edema post-stroke* (42).

While there are several studies that demonstrated the prognostic value of copeptin in acute ischemic stroke patients, there are only 4 studies that took into consideration recanalization therapies (4, 37, 43, 44). The efficacy of recanalization therapies are time-dependent and change the prognostic of patients, so fast diagnosis of ischemic stroke is needed (15).

This review summarizes and discusses the value of copeptin in ischemic stroke *diagnosis*, short-term and long-term *prognosis* of ischemic stroke and its importance in relation with *treatment options*. The present study presents a review of recent studies that assessed: (1) diagnostic and prognostic value of plasma copeptin concentration in acute ischemic stroke patients; (2) prognostic value of plasma copeptin concentration in acute ischemic stroke patients who were eligible for recanalisation therapies.

## 2 Methodology

We performed a review of the literature analyzing PubMed and Scopus databases. We used “stroke” AND “copeptin” as keywords. The search was limited to articles in English language. We applied the search filter of publications between the years 2010–2024. We analyzed only available full-text articles, such as clinical trials and randomized clinical trials, excluding reviews, systematic reviews and meta-analyses. PRISMA criteria were used.

Thus, the PubMed database revealed 141 results. We excluded articles that were reviews, systematic reviews or meta-analysis (*n* = 32). We excluded studies performed *exclusively on transient ischemic attack* (*n* = 4). We excluded studies performed *exclusively on subarachnoid hemorrhage* (*n* = 5). We excluded studies performed *exclusively on intracerebral hemorrhage* (*n* = 4). We excluded articles that focused on copeptin as a *marker for differential diagnosis* (*with vertigo, with stroke mimics, between stroke subtypes; n* = 6). We excluded studies that evaluated *exclusively other biomarkers, not copeptin* (*n* = 3). We excluded studies that evaluated patients with diagnoses other than stroke or symptoms suggestive of another condition (end-stage renal disease, chronic renal disease, adult polycystic kidney disease, chest pain, acute myocardial infarction, heart failure, stable coronary disease, atrial fibrillation, coronary artery ectasia, pulmonary embolism, pregnancy, cirrhosis, craniopharyngioma, chronic insomnia, post-stroke depression, post-stroke infections, hyponatriemia/hypernatriemia; *n* = 50). We excluded studies that were performed on non-acute stroke patients (patients recovering from stroke, patients undergoing non-cardiac surgery, patients after coronary surgery, patients after elective carotid endarterectomy, recreational marathon runners, healthy people, elderly people, dialysis patients; *n* = 9). We excluded nursing practice guidelines (*n* = 1). We excluded one more article that was in Chinese language (*n* = 1). In the end, PubMed database revealed 26 results.

The Scopus database revealed 191 results. After applying the criteria (article types, English language only), 131 relevant articles were identified. We excluded articles that were reviews or meta-analysis (*n* = 9). We excluded studies performed *exclusively on transient ischemic attack* (*n* = 3). We excluded studies performed *exclusively on subarahnoid hemorrhage* (*n* = 3). We excluded studies performed *exclusively on intracerebral hemorrhage* (*n* = 4). We excluded articles that focused on copeptin as a *marker for differential diagnostic* (*with vertigo, with stroke mimics, between stroke subtypes; n* = 6). We excluded studies that evaluated *other biomarkers*, but not copeptin (*n* = 3). We excluded studies that evaluated other biomarkers, but not copeptin (*n* = 2). We excluded studies that evaluated patients with other diagnosis than stroke (patients with acute illnesses, hyperthyroidism, acute myocardial infarction, cardiogenic shock, pulmonary embolism, heart failure with preserved ejection fraction, acute coronary syndrome, atrial fibrillation, heart failure with reduced ejection fraction, preeclampsia, chronic heart failure, coronary artery ectasia, chronic obstructive pulmonary disease, chronic insomnia, chest pain, cirrhosis, polycystic kidney disease, post stroke depression, post-stroke infections, post-stroke fever, pregnancy, resynchronization therapy, hypernatriemia/hyponatriemia, acute illness, acute mental stress; *n* = 55). We excluded studies that were performed on non-acute stroke patients (patients who recovered from stroke, patients after coronary surgery, patients after cardiac surgery, patients undergoing non-cardiac surgery, patient undergoing elective carotid endarterectomy, recreational marathon runners, healthy people, elderly people, dialysis patients; *n* = 9). We excluded articles that were only protocols or nursing practice guidelines (*n* = 1). We excluded a study performed on animals (*n* = 1). In the end, Scopus database revealed 29 results.

After excluding duplicate articles between the two databases (*n* = 24), 31 articles resulted for study. The number of patients included in the analyzed studies varied between 18 and 4,302.

**Figure 1:**
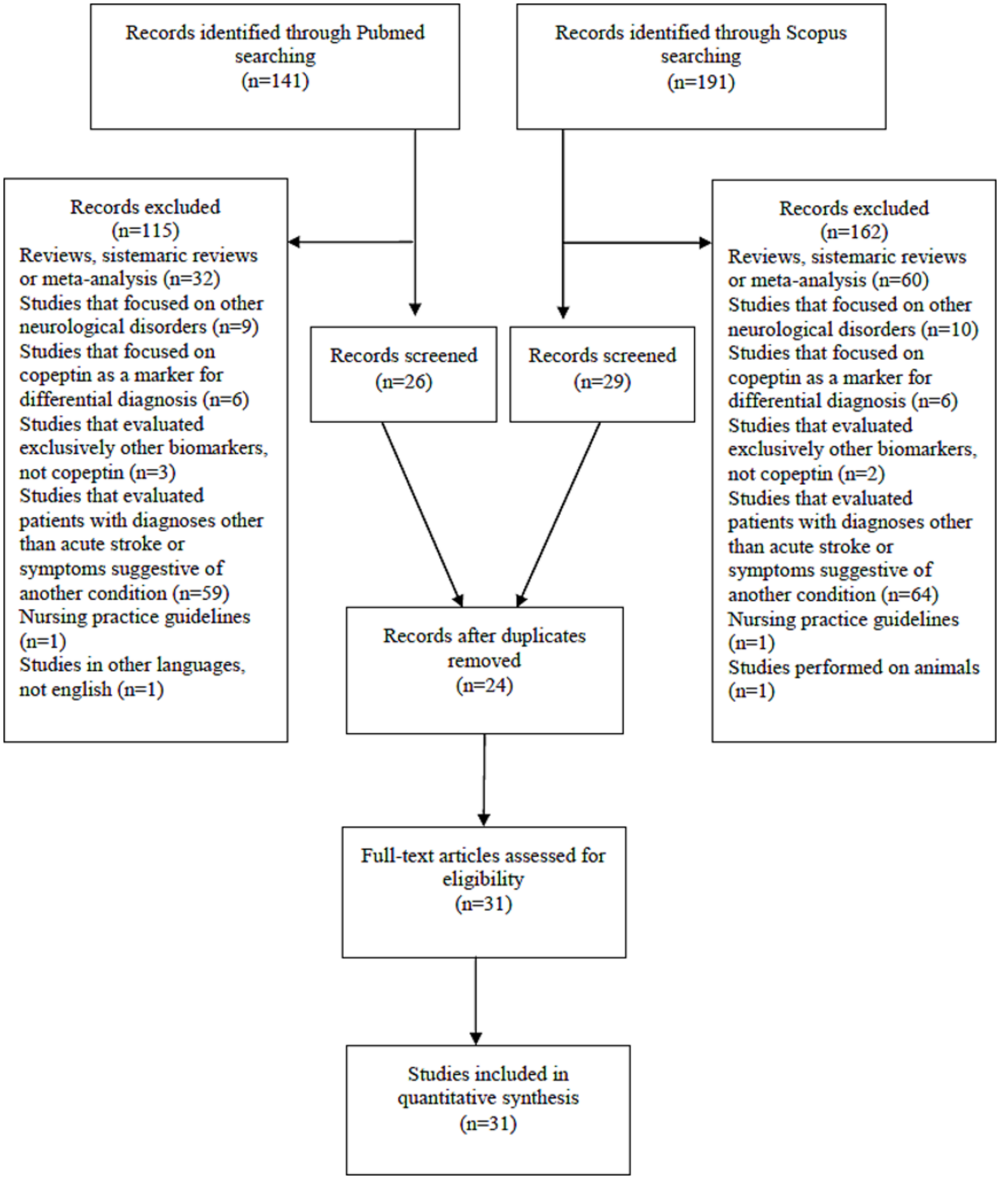
PRISMA flow diagram.

The PRISMA flow diagram that summarized the screening process can be seen in Figure 1.

## 3 Results

### 3.1 Copeptin as a prognostic biomarker in acute ischemic stroke

Jihad et al. (45) evaluated renalase, copeptin, N-terminal pro B-type natriuretic peptide (NT-proBNP) and MMP-9 concentrations in 42 ischemic stroke patients in comparison to 40 healthy individuals. They demonstrated a significant increase in the levels of copeptin, NT-proBNP and MMP-9 for stroke patients compared to healthy controls. Moreover, they also found a significant difference according to severity for stroke patients for copeptin (0.0012), renalase (0.0069) and MMP-9 (0.0094): for severe ischemic stroke (National Institutes of Health Stroke Scale (NIHSS) score > 14) compared to moderate ischemic stroke (NIHSS score < 14).

The results of a study by Keshk et al. (46) suggest that copeptin, thrombomodulin, and alarmin signaling pathways play an important role in the chronic inflammatory state of obese patients with ischemic stroke. The study included 30 ischemic stroke patients (15 overweight and 15 normal weight) and compared them to 20 healthy controls (10 overweight and 10 normal weight). Their result suggested that copeptin level was significantly increased in overweight ischemic stroke patients with NIHSS scores over 7 points. They found increased copeptin levels to all overweight stroke patients. Also, copeptin levels correlated with anthropometric measurements, severity scores and vascular damage scores. The authors hypothesized that increased inflammation and endogenous stress in obesity and after an ischemic stroke stimulate the same hypothalamo-hypophysis-vasoppresin axis (46). Similar to previous studies, they suggested that higher copeptin levels are linked to obesity, metabolic syndrome and insulin resistance (47).

Wannamethee et al. (48) reported that copeptin is independently associated with an increased risk of stroke and cardiovascular mortality in men with diabetes. They conducted a prospective study on 3,536 men aged between 60 and 79 years old who were followed to evaluate the occurrence of myocardial infarction, stroke events and cardiovascular disease deaths. They demonstrated that there is no association between copeptin and incident stroke in men *without* diabetes, while *elevated copeptin levels were associated with increased risk of stroke* and cardiovascular disease mortality in men *with* diabetes. Their result suggested that targeting the AVP system might have beneficial effects on cardiovascular disease mortality and stroke risk in older men with diabetes (48). Their findings demonstrate that *copeptin is associated with the development of stroke, even in diabetic patients without prevalent stroke*. It is still uncertain if there is a causal relationship between copeptin and stroke risk in patients with diabetes, but it was not explained by conventional risk factors such as stroke, insulin resistance or level of NT-proBNP. Studies have shown that copeptin is strongly associated with microalbuminuria, suggesting the role of AVP system in producing albuminuria, which has been linked to incident stroke in diabetic patients (49, 50). This may be the consequence of the antidiuretic effects of vasopressin, leading to increased excretion of albumin (51). Therefore, the relationship between copeptin levels and incident stroke may be linked to the development of albuminuria (48). This conclusion is important in relation to treatment options, such as empagliflozin. This molecule was added to the standard treatment protocol for reducing morbidity and mortality in patients with type 2 diabetes at high cardiovascular risk (48). Empagliflozin is a sodium glucose cotransporter (SGLT)-2 inhibitor that stimulates the excretion of glucose leading to osmotic diuresis and reduced blood pressure.

Fenske et al. (52) analyzed data from hemodialysis patients with type 2 diabetes and reported that copeptin levels strongly associated with an increased risk of stroke, sudden death, cardiovascular events and mortality.

Katan et al. (53) evaluated several blood markers in stroke patients: procalcitonin, copeptin and MRproANP. They evaluated 3,298 healthy individuals who were followed-up. During the follow-up period, 172 individuals developed acute ischemic stroke. The authors compared their results with 344 healthy controls. In their unadjusted analysis, individuals who were in *the top copeptin quartile had an increased risk of ischemic stroke compared to those in the lowest quartile*. However, they did not report any significant association between copeptin and stroke etiology. Katan et al. (53) support the association between ischemic stroke and procalcitonin and MRproANP. The associations of procalcitonin and MRproANP differed by stroke etiology based on TOAST classification. Procalcitonin levels in the top quartile were associated more with small vessel stroke and MRproANP levels were associated with cardioembolic stroke.

Perovic et al. (54) assessed differences in resistin and copeptin concentration between 112 ischemic stroke patients and 63 healthy controls, but also assessed whether these markers have any prognostic value for *ischemic volume*, *stroke severity* (measured by NIHSS score) and *patients’ functionality* (measured by Barthel Index). Resistin is an adipocytokine produced by adipocytes that has been shown to be a promising prognostic marker in lacunar ischemic stroke (55). Perovic et al. (54) mentioned that the increase in resistin levels in the acute phase after stroke may be explained by an early inflammatory response to acute tissue injury. However, studies are contradictory regarding the role of resistin in stroke. Weikert et al. (56) reported that elevated levels of resistin are associated with a higher risk of myocardial infarction but not with stroke risk. Perovic et al. (54) demonstrated that resistin levels were significantly higher in stroke patients compared to healthy controls (3.2 mg/L versus 2.5 mg/L), but found no correlations between resistin level and NIHSS score, Barthel index or ischemic volume. On the other hand, copeptin concentration did not differ between healthy controls and stroke patients. However, *among stroke patients, copeptin levels differentiated those with good functionality from those with poor functionality* (Barthel index below 60), with higher levels observed in patients with a lower index. This result suggests that copeptin can predict the functionality of patients. The researchers did not identify differences in copeptin levels between groups based on NIHSS severity score. They also disproved the hypothesis that resistin and copeptin levels vary with infarction volume. The conclusion of Perovic et al. (54) was that resistin, but not copeptin, is elevated in ischemic stroke patients compared to age and sex matched healthy controls.

Hotter et al. (1) demonstrated an association between NIHSS score and IL-6, as well as an association between 90-day functional outcomes (measured with the mRS) and levels of copeptin, MR-proADM, IL-6, and HLA-DR in their evaluation of 91 stroke patients. IL-6 was the only biomarker that correlated with some radiological features (acute volume of DWI, extent of perfusion imaging (PI), DWI-PI-mismatch, final lesion volume, cortical infarcts). Their conclusion support the idea that IL-6 is an inflammatory marker of cerebral parenchymal damage and that copeptin correlated with functionality at 3 months (1).

Oraby et al. (43) evaluated 45 patients with first ever acute ischemic stroke and 45 healthy controls in order to investigate the relationship between copeptin and short-term prognosis of acute ischemic stroke after 3 months (measured by mRS). They demonstrated that copeptin levels were higher in stroke patients (mean of copeptin 120.52 pg./mL), compared to healthy controls (mean of copeptin 76.51 pg./mL). In addition, patients with severe clinical presentation (NIHSS>16 points) had a higher level of copeptin (mean of copeptin 139.45 pg./mL) in comparison to those with mild-to-moderate stroke (NIHSS 0–15 points; mean of copeptin 95.47 pg./mL). Patients with unfavorable outcome (mRS > 3) had a higher level of copeptin (mean of copeptin 142.45 pg./mL) in comparison to those with favorable outcome (mRS 0–2 points; mean of copeptin 93.47 pg./mL). Another important finding was that copeptin was significantly lower in patients who were eligible for revascularization with recombinant tissue plasminogen activator (rTPA; mean of copeptin 99.55 pg./mL compared to 127.31 pg./mL). They did not identify any significant differences between copeptin levels and ischemic stroke subtypes according to TOAST classification. However, they reported that patients with diabetes mellitus, hypertension and dyslipidemia had a higher levels of copeptin. Other significant correlation identified were with: volume of infarction measured by MRI, NIHSS score, clinical outcome at 3 month follow-up.

Hotter et al. (6) analyzed 573 stroke patients and evaluated several biomarkers (procalcitonin, copeptin, C-terminal pro-endothelin and midregional pro-adrenomedullin) and demonstrated an association between all of them and functional outcome at 3 months and death. Another interesting result was that cardioembolic stroke was associated with higher levels for all biomarkers, especially for MRproANP. However, none of the biomarkers improve the prediction of death or functional outcome in regression models. The multivariate model for predicting death at 90 days retained only age and NIHSS as predictors.

In a study evaluating stroke in patients with type 2 diabetes, researchers evaluated the prognostic value of copeptin on patients’ functionality and mortality at 3 months (57). They evaluated 247 patients, and the median copeptin value was 14.3 pmol/L. Copeptin level was strongly associated with unfavorable functional outcome. Copeptin levels increased with stroke severity, as defined by the NIHSS score. In the group of patients who underwent cerebral MRI, copeptin levels were associated with lesion size on MRI: for small lesions (<10 mL), the copeptin level was 6.9 pmol/L; for medium-sized lesions (10–100 mL), the level was 13.6 pmol/L; and for large lesions (>100 mL), the level was 18.1 pmol/L. At 3 months, 86 patients (34.8%) had favorable functionality. Copeptin level in patients with unfavorable functionality was significantly higher compared to those with favorable functionality (16.2 pmol/L compared to 12.4 pmol/L). In addition, among the 41 patients who died, *copeptin levels were almost twice as high compared to those who survived* (20.9 pmol/L vs. 12.4 pmol/L). Their main finding was that copeptin predicts the development of unfavorable functional outcome independent of NIHSS score, FBG or CRP. Thus, the conclusion of Wang et al. (57) was that an increased level of copeptin plays an important role in the progression of stroke in patients with comorbid type 2 diabetes.

De Marchis et al. (37) evaluated 783 patients with acute ischemic stroke and demonstrated that copeptin independently predicts unfavorable outcome [modified Rankin Score (mRS) > 3 points] within 90 days, mortality within 90 days and complications (symptomatic intracerebral hemorrhage, space-occupying cerebral edema, pneumonia, seizures or mortality within 10 days from stroke onset). They concluded that copeptin is a validated blood marker that adds predictive value for outcome and mortality at 3-month follow-up beyond stroke severity and age.

De Marchis et al. (58) developed a score (CoRisk score) that can predict the 3 month poor prognosis of patients with ischemic stroke (measured by mRS). The study was conducted on 1,102 patients. The score is calculated based on copeptin level, age, NIHSS score, and recanalization therapy. The advantage of CoRisk score is that it adds the recanalization therapy, because the most prominent current prognostic scales are lacking variables for acute treatment (59). Moreover, evidence shows that eligibility for acute treatment improves outcomes for stroke patients, and the importance of including it in prognosis scores is well-established (14). They demonstrated that age, NIHSS score, and copeptin were independent variables associated with 3-month prognosis and mortality. Then, the researchers evaluated two prediction models: the first predicted functional prognosis using the mRS score; the second predicted death versus survival at 3 months post-stroke. An unfavorable prognosis was identified in 436 (40%) of the patients, and the CoRisk score correctly classified 75% of patients. The CoRisk score demonstrated a sensitivity of 67% and a specificity of 80% in predicting prognosis. For predicting death versus survival, their model was not well calibrated. De Marchis et al. (58) acknowledged that a limitation of their study was testing in a limited geographic area and emphasized the importance of validating the CoRisk score in other regions. Second limitation of their study was the lack of validation for mortality prediction. However, the main strength of CoRisk score was that it has a high prognostic accuracy despite having only 4 variables, all easily accessible in the emergency room, only using blood sampling (58).

Urwyler et al. (60) demonstrated on 362 patients that copeptin was an independent predictor of functional outcome after 1 year (measured by mRs) and all-cause mortality. In the 146 patients with poor functional outcome, copeptin levels were higher compared to the patients with good functional outcome (19.3 pmol/L compared to 8.12 pmol/L). In their regression analysis, the authors demonstrated that copeptin had a better prediction for prognosis than white blood cell count, CRP and glucose, but a similar prediction rate to NIHSS score. In terms of analyzing mortality rate, copeptin levels in nonsurvivors were higher compared to survivors (28.10 pmol/L compared to 9.34 pmol/L). In their regression analysis, the authors demonstrated that copeptin had a similar prediction rate to NIHSS score in predicting mortality, but better results in comparison to white blood cell count, CRP or glucose. Urwyler et al. (60) concluded that copeptin may be a reliable and independent marker for predicting long-term outcome in ischemic stroke patients. They demonstrated that copeptin is a better prognostic marker compared to other markers, because it improves the prognostic value of the NIHSS score for long-term mortality and functional outcome at 1-year of follow-up. Compared to other brain markers, copeptin mirrors intracerebral processes and is released directly into circulation, bypassing the blood–brain barrier (60).

Zhang et al. (27) evaluated 245 ischemic stroke patients and 100 healthy volunteers in order to investigate the relationship between copeptin levels and functional outcome (mRS) and mortality within 1 year. Patients with stroke had a higher level of copeptin compared to control cases (12.4 pmol/mL compare to 3.9 pmol/mL). Patients with unfavorable outcome and increased levels of copeptin at admission compared to those with favorable outcome (21.9 pmol/mL versus 10.3 pmol/mL). Copeptin levels increased with the increase on the NIHSS scale: the mean copeptin for patients with NIHSS 0–6 points was 8.3 pmol/mL, for patients with NIHSS 7–15 points was 14.3 pmol/mL, for patients with NIHSS >15 points was 27.2 pmol/mL. Copeptin levels increased with the increased lesion size on MRI: the mean copeptin for patients with small lesions was 6.2 pmol/mL, for patients with medium lesions was 13.9 pmol/mL, for patients with large lesions was 17.9 pmol/mL. Also, non-survivors had a higher level of copeptin at admission (mean copeptin for patients who died was 36.2 pmol/mL versus 10.6 pmol/mL for patients who survived). Their multivariate regression showed that copeptin was an independent predictor for functional outcome and survival.

Tu et al. (61) conducted a multicenter observational study of 4,125 patients, where they demonstrated that copeptin and NT-proBNP levels in patients with ischemic stroke can predict all-cause mortality and cardiovascular mortality at 1 year. In the follow-up year, 906 patients (20.1%) died, of which 589 died from cardiovascular causes (13.1%). They revealed a significant correlation of copeptin level with age, glomerular filtration rate, NIHSS score and NT-proBNP level. For patients in whom brain MRI was available, the *volume of the infarction correlated with copeptin levels*. Also, patients with *previously known type 2 diabetes were those with higher levels of copeptin*. During 1 year follow-up, Tu et al. (61) demonstrated that both NT-proBNP and copeptin are independent prognostic biomarkers for assessment of prognosis of stroke. Tu et al. (61) demonstrated an association between the level of the two biomarkers and mortality, managing to stratify the research group into 3 risk groups: those with low risk (those with the two biomarkers below average), those with intermediate risk (with one of the two biomarkers above average) and those at increased risk (with both markers above average). Tu et al. (61) speculated on the possible mechanisms underlying their results: AVP secretion may be stimulated through the brainstem and limbic system, triggered by stress factors. Thus, the concentration variation of copeptin may be useful for evaluating the severity of damage independently of lesion dimensions, age, sex and clinical impairment at the moment of admission. Another mechanism speculated was that ischemic neuronal injury stimulates adrenocorticotropic hormone that leads to hypercortisolism and may influence the level of copeptin/AVP (61).

Tang et al. (4) evaluated 316 patients with ischemic stroke and wanted to determine copeptin levels and its association with stroke recurrence during the first year of follow-up. Patients were followed at 3, 6 and 12 months after the stroke. Recurrence of stroke was defined as the sudden functional deterioration of the neurological status demonstrated by an increase in the NIHSS score by at least 4 points or by having a new focal neurological deficit lasting more than 4 h. One of the results of the study was that 54 patients (17.1%) had a *stroke recurrence* and in these patients copeptin levels were *higher compared to those without recurrence* (28.9 pmol/L versus 21.0 pmol/L). 135 patients in their study had a *minor stroke* (NIHSS score below 5 points) and they had a *lower copeptin level compared to those with more severe forms* (19.5 pmol/L versus 25.4 pmol/L). The researchers demonstrated that copeptin levels above 31.8 pmol/L were associated with a NIHSS score above 6 points. The positive correlation between copeptin levels and moderate-to-severe clinical severity demonstrates that *copeptin mirrors the stress associated with extensive stroke*. They demonstrated positive correlations between copeptin and cortisol levels, infarcted brain volume, body mass index, age, systolic blood pressure (BP), diastolic BP and NIHSS score. One of the most important conclusions of the study was that copeptin, age, NIHSS score, infarct volume, stroke etiology, revascularization treatment, personal history of atrial fibrillation, CRP, homocysteine and cortisol levels are *predictors of stroke recurrence*. In addition, they demonstrated that copeptin has a higher predictive value for stroke recurrence than CRP, homocysteine, cortisol, or NIHSS score. Another interesting result of their study was that the median copeptin levels were higher for atherothrombotic stroke subtype compared to other stroke subtypes. The researchers concluded that an increased level of copeptin plays an important role in the progression of ischemic stroke. Tang et al. (4) concluded that copeptin is an important and independent marker that predicts 1-year stroke recurrence in patients with ischemic stroke. Moreover, they demonstrated that copeptin can improve the prognostic value of NIHSS score.

Greisenegger et al. (23) reported that, after adjusting for age, sex and risk factors, copeptin predicted recurrent vascular events, recurrent ischemic stroke and death, particularly after cardioembolic TIA/stroke. Accurate prediction of recurrent vascular event is extremely important, because patients at high risk should benefit from more aggressive secondary preventive strategies and should be included in trials of new treatments (23). Interestingly, in their study, levels of copeptin did not differ statistically significant between first sampling and 1-year follow-up with a median of 6.0 pmol/L and 5.4 pmol/L, respectively. Moreover, the researchers detected a significant association between plasma copeptin levels and etiology of stroke and a borderline interaction with sex: the predictive value of copeptin was more pronounced in male patients and in patients with cardioembolic stroke. Another important aspect reported by Greisenegger et al. (23) was *comparing* the predictive value of copeptin for recurrent vascular events *with other standard biomakers related to: inflammation* (CRP, IL-6, Neutrophil gelatinase associated lipocalin, Tumor necrosis factor receptor 1), *thrombosis* (von Willenbrand factor, D-dimer, P-selectin, FBG, Thrombomodulin, protein-Z), *cardiac or neuronal function or injury* (Heart-type fatty acid binding protein, NT proBNP, Neuron specific enolase, Brain derived neurotrophic factor). They demonstrated that copeptin had the highest predictive value in the prognostic of recurrent vascular events and exceeded all other biomarkers (23). Their conclusion was that copeptin might help with better risk stratification of patients with stroke.

Zeng et al. (62) evaluated 4,302 ischemic stroke patients in a multicenter cohort study to determine whether plasma copeptin and NT-proBNP levels correlated with stroke recurrence within 3 months of the initial event. In their receiver-operating characteristic analysis of stroke recurrence, the authors demonstrated an increase from 0.80 to 0.83 when adding NT-proBNP to clinical scores and an increase from 0.83 to 0.86 when adding both NT-proBNP and copeptin levels to clinical scores. They demonstrated that copeptin levels may predict stroke recurrence, especially for patients with higher than median NT-proBNP levels. Another interesting idea suggested by Zeng et al. (62) was that the fact that copeptin and NT-proBNP have complementary power may suggest that these two markers are stimulated by different aspects of cardiovascular homeostasis. Their complementary power also explain that both markers together may be useful for risk stratification in stroke patients, more than each marker alone (62).

Wang et al. (63) evaluated the correlation between copeptin levels and 1 year mortality on 275 ischemic stroke patients which were recruited within 24 h after onset. Copeptin levels were significantly higher in acute ischemic stroke patient compared to healthy controls. They found a significant correlation between copeptin levels and NIHSS score. Infarction lesions on MRI correlated with copeptin levels (for small lesions 6.8 pmol/L, for medium lesions 15.9 pmol/L, and for large lesions 20.2 pmol/L). Moreover, the authors found that copeptin levels were higher in patient with atherosclerosis subtype of ischemic stroke. The authors observed that copeptin levels were significantly higher in non-survivors compared to survivors. They also found copeptin as an independent stroke mortality predictor, which an area under ROC curve of 0.882, demonstrating a sensitivity of 90.7% and specificity of 84.5% (63). The cut off value for copeptin as an indicator or mortality was around 20.5 pmol/L. In their multivariate analysis, the predictors for death were: copeptin levels, NIHSS score, age, CRP, D-dimer.

Similar to Wang et al. (63), Katan et al. (64) analyzed 362 patients with ischemic stroke and demonstrated that copeptin levels positively associated with unfavorable outcome and mortality within 90 days. Copeptin levels positively correlated with NIHSS scores and mRS scores and paralleled infarct lesion sizes on MRI. Copeptin levels were more than 3 times greater in patients who died compared to survivors. In their univariate analysis for predicting functional outcome, the significant variables were: copeptin, CRP, age, female sex, NIHSS, hypertension, atrial fibrillation, total anterior circulation syndrome, posterior circulation syndrome and small vessel occlusive. In their univariate analysis for predicting mortality within 90 days, the significant variables were: copeptin, CRP, glucose, age, NIHSS, atrial fibrillation, coronary heart disease, total anterior circulation syndrome and small-vessel occlusive. The authors demonstrated that the predictive value of copeptin was comparable with the predictive value of NIHSS score, but superior to CRP and glucose (64). Their conclusion was that copeptin had a prognostic accuracy that is superior to other commonly used laboratory parameters or clinical variables.

In contrast, Richard et al. (65) evaluated several biomarkers (4 cell adhesion molecules, CRP, IL-6, NT-proBNP, troponin, copeptin and S100 calcium binding protein B) on patients presenting with ischemic stroke, hemorrhagic stroke and transient ischemic attack. They did not find any association with copeptin levels.

Table 1 presents a summary of findings on copeptin as a prognostic biomarker in acute ischemic stroke, highlighting the number and types of patients included, assessed biomarkers, evaluated outcomes (stroke severity, stroke occurrence, short-term and long-term prognosis, scales used, stroke recurrence, and mortality), findings (correlations and predictions), and practical applications.

**Table 1:** Studies that evaluated copeptin as a prognostic biomarker in acute ischemic stroke.

Study	Participants	Assessed biomarkers	Consequence	Finding (associations and predictions)	Practical application
Jihad et al. (45)	42 stroke patients versus 40 healthy controls	Copeptin Renalase MMP-9 NT-proBNP	Stroke severity	Elevated copeptin levels correlate with stroke severity (NIHSS)	
Zhou et al. (68)	70 stroke patients versus 40 healthy controls	Copeptin	Stroke severity	Copeptin levels were higher in patients with cerebral infarction and intracerebral hemorrhage compared to healthy subjects Copeptin levels positively correlated with NISS score and mRS score	Copeptin level has a certain value in the clinical diagnosis and prognosis of stroke
Keshk et al. (46)	50 ischemic stroke patients (15 overweight and 15 normal weight) versus 20 healthy controls (10 overweight and 10 normal weight)	Copeptin Thrombomodulin High mobility group box1 Lipocalin 2	Stroke severity	Copeptin level was significantly increased in overweight ischemic stroke patients with NIHSS scores over 7 points	Copeptin is an acute damage marker for ischemic stroke patients Copeptin may play a role in central adiposity that leads to metabolic syndrome
Wannamethee et al. (48)	3,536 men with diabetes	Copeptin	Stroke occurrence during follow-up period (approx. 13 years)	Elevated copeptin levels were associated with increased risk of stroke and cardiovascular disease only for men with diabetes	Copeptin is associated with development of stroke, even in diabetic patients without prevalent stroke Need to control diabetic risk factors Treatment options such as empagliflozin for patients with high cardiovascular risk
Katan et al. (53)	172 acute ischemic stroke patients versus 344 healthy controls	Copeptin Procalcitonin MR-proANP	Stroke occurrence in the follow-up period (approx. 13 years)	Individuals who were in the top copeptin quartile had an increased risk of ischemic stroke compared to those in the lowest quartile	Other markers (procalcitonin and MR-proANP) are associated with ischemic stroke risk
Perovic et al. (54)	112 acute ischemic stroke patients versus 63 healthy controls	Copeptin Resitin	Functionality at dischange (Barthel index)	Copeptin made no difference between stroke patients and healthy controls Copeptin was higher among patient with poor functionality (Barthel index<60) No differences in copeptin in terms of NIHSS or lesion size	
Dong et al. (69)	125 acute ischemic stroke patients	Copeptin	Functionality at 90 days (mRS)	Copeptin positively correlated with NIHSS score Copeptin levels were higher in patients that had a poor functional outcome, compared to those with favorable outcome Copeptin levels were almost double for patients who died compared to those who survived Copeptin was an independent predictor for poor functional outcome and mortality	
Hotter et al. (1)	91 acute ischemic stroke patients	Copeptin MR-proADM IL-6 HLA-DR	Functionality at 90 days (mRS) Radiologic features of the stroke lesion	There is an association between copeptin and functionality at 90 days Only IL-6 correlated with some radiological features of stroke lesion	No association between copeptin and radiologic features of the lesion
Oraby et al. (43)	45 acute ischemic stroke patients versus 45 healthy controls	Copeptin	Functionality at 3 months (mRS)	Copeptin levels were higher among stroke patients compared to healthy controls Patients with severe clinical presentation had a higher level of copeptin compared to those with mild-to-moderate stroke Patients with unfavorable outcome (mRS > 3) had a higher level of copeptin compared to those with favorable outcome (mRS 0–2 points) Copeptin was significantly lower in patients who were eligible for revascularization with rTPA No differences in copeptin levels according to TOAST classification Patients with diabetes, HTA and dyslipidemia had higher levels of copeptin Copeptin correlated with lesion size of MRI	
Hotter et al. (6)	573 acute ischemic stroke patients	Copeptin Procalcitonine C-terminal pro-endothelin Midregional pro-adrenomedullin	Functionality at 3 months Mortality at 3 months	All biomarkers were associated with functional outcome and mortality Cardioembolic strokes were associated with higher levels of all biomarkers None of the biomarkers did not improve the prediction of death or functional outcome, the only predictors remaining age and NIHSS	
Wang et al. (57)	247 acute ischemic stroke patients with diabetes	Copeptin Fibrinogen CRP	Functionality at 3 months (mRS) Mortality at 3 months	Copeptin levels in patients with poor functionality was higher compared to those with good functionality Copeptin correlates with NIHSS, lesion size Copeptin levels were 2 times higher in patients who died, compared to those who survived	Copeptin predict a poor prognosis intendent of NIHSS, FBG or CRP Copeptin plays an important role in the progression of stroke patients with type 2 diabetes comorbidity
De Marchis et al. (37)	783 acute ischemic stroke patients	Copeptin	Functionality at 3 months (mRS) Mortality at 3 months Complications	Copeptin independently predicts poor outcome within 3 months, mortality within 3 months and complications (symptomatic intracerebral hemorrhage, space-occupying cerebral edema, pneumonia, seizures or mortality within 10 days from stroke onset)	
De Marhis et al. (58)	1,102 acute ischemic stroke patients	Copeptin	Functionality at 3 months (mRS)	Age, NIHSS and copeptin levels were independent variables associated with 3-month functionality	CoRisk score that consist of: copeptin, age, NIHH, recanalisation therapy
Urwyler et al. (60)	362 ischemic stroke patients	Copeptin	Functionality at 1 year (mRS) All-cause mortality	Patients with poot functionality had higher levels of copeptin Copeptin in non-survivors were higher than in survivors Copeptin predicted better than WBC, CRP and glucose, but similar to NIHSS	Copeptin is a reliable marker for long-term prognosis
Zhang et al. (27)	245 acute ischemic stroke patients versus 100 healthy controls	Copeptin	Functionality at 1 year (mRS) Mortality at 1 year	Stroke patients has higher levels of copeptin compared to healthy controls Patients with poor functionality had higher levels of copeptin compared to those with good functionality Copeptin levels increased with the increase of NIHSS Non-survivors had a higher level of copeptin compared to survivors	
Wang et al. (63)	275 ischemic stroke patients versus 100 healthy controls	Copeptin	Mortality at 1 year	COPEPTIN levels were significantly higher in acute ischemic stroke patient compared to healthy controls Significant correlation between copeptin levels and NIHSS score INFARCTION lesions on MRI correlated with copeptin levels—copeptin levels were higher in patient with atherosclerosis subtype of ischemic stroke. Copeptin levels were significantly higher in non-survivors compared to survivors. Copeptin is an independent stroke mortality predictor, demonstrating a sensitivity of 90.7% and specificity of 84.5% at a cut-off value of 20.5 pmoL/L Predictors for 1 year mortality were: copeptin, NIHSS score, age, CRP and D-dimer	
Tu et al. (61)	4,125 acute ischemic stroke patients	Copeptin NT-proBNP	All-cause mortality at 1 year Cardiovascular mortality at 1 year Risk stratification	Copeptin correlated with age, glomerular filtration rate, NIHSS, NT-proBNP level and lesion size Higher levels of copeptin occured at diabetic patients Both copeptin and NT-proBNP are independent prognostic biomarkers for prognosis	Stratify stroke patients into 3 risk group according to levels of copeptin and NT-proBNP: low risk (both biomarkers below average), those with intermediate risk (with one of the two biomarkers above average) and those at increased risk (both markers above average)
Zeng et al. (62)	4,302 acute ischemic stroke patients	Copeptin NT-proBNP	Stroke recurrence during 3 months follow-up	Copeptin levels predict stroke reccurence, especially for patients with higher than median NT-proBNP levels	Copeptin alongside NT-proBNP may help for risk stratification in acute ischemic stroke patients, more than each marker alone
Tang et al. (4)	316 acute ischemic stroke patients	Copeptin Cortisol Homocysteine	Stroke recurrence during 1 year follow-up	Patients who had a stroke recurrence were the ones with higher levels of copeptin Patients with more severe stroke forms (NIHSS>5) had higher levels of copeptin Median copeptin levels were higher for atherosclerosis stroke subtype Copeptin, age, NIHSS, infarct volume, stroke etiology, revascularisation treatment, personal history of atrial fibrillation, CRP, homocysteine and cortisol levels are predictors of stroke recurrence.	Copeptin mirrors the stress associated with extensive stroke
Greisenegger et al. (23)	1,076 patients with acute ischemic stroke or TIA	Copeptin CRP IL-6 Tumor necrosis factor receptor 1 Neutrophil gelatinase associated lipocalin von Willenbrand factor D-dimer P-selectin Fibrinogen Thrombomodulin protein-Z Heart-type fatty acid binding protein NTproBNP Neuron specific enolase Brain derived neurotrophic factor	Reccurence of vascular event during the follow-up period (approx. 12 years)	The predictive value of copeptin was more pronounced in male patients and in patients with cardioembolic stroke Copeptin has the highest predictive value in predicting recurrent vascular events	Copeptin may help for a better risk stratification in terms of reccurence Patients at high risk for recurrence should profit from more aggressive secondary preventive strategies

### 3.2 Copeptin in acute ischemic stroke depending on revascularization strategies

Several studies have suggested that prognostic scores for ischemic stroke patients need to consider intravenous or endovascular recanalization therapies (14). Many studies proved the relation between copeptin and functional outcome and mortality in ischemic stroke patients, but Spagnolello et al. (44) is the first one that added the relation to acute intervention and complications of stroke (cerebral edema and hemorrhagic transformation).

Spagnolello et al. (44) evaluated the temporal profile of copeptin levels in revascularized ischemic stroke patients and sought to identify correlations with the development of cerebral edema and hemorrhagic transformation. Researchers evaluated 34 patients at the time of admission (T0), 24 h post-recanalization procedure (T1), and 3–5 days after admission (T2) with imaging and serologic evaluations.

They demonstrated a mean copeptin concentration of 50.71 pmol/L at time T0; 18.31 pmol/L at time T1; and 10.92 pmol/L at time T2. The researchers found no correlation between copeptin levels and the length of time since the onset of symptoms. Copeptin levels at the *time of admission (T0) were higher in those with NIHSS score above 10 points*. In addition, although not statistically significant, *higher* levels of copeptin were identified in patients who were *hospitalized in less than 6 h* after the onset of symptoms. Copeptin levels at T0 correlated with a worse functional outcome at 1-year follow-up. Copeptin levels at T1 correlated with worse functional outcome at 1-year follow-up and mortality at 1-year follow-up. Copeptin levels at T2 were higher at patients with mRS over 3 points or died at the 1-year follow-up (44).

When analyzing the complications of stroke, the authors demonstrated that copeptin level at time T1 was more elevated in patients who, subsequently, at time T2, had moderate–severe brain edema. In addition, patients with hemorrhagic transformation at T1 and T2 were shown to have increased levels of copeptin at T1 (44).

When analyzing the relation to revascularization therapy, the authors highlighted that: the difference in copeptin level between T1 and T0 was significantly greater in patients who received revascularization therapy, especially in those who received *both* thrombolysis and thrombectomy compared with those who received only conservative management. Differences in copeptin between T1 and T0 were: −4.50 pmol/L in patients who received only thrombolysis; −37.84 pmol/L in patients who received only thrombectomy; −129.34 pmol/L in patients who received thrombolysis and thrombectomy. In addition, the researchers also demonstrated a positive correlation between the *difference in copeptin levels between T1 and T0* with the *Thrombolysis in Cerebral Infarction (TICI) score* (44).

The first conclusion of their study was that copeptin is associated with resolution of cerebral edema and hemorrhagic transformation in ischemic stroke. The second conclusion of their study was that the decrease in copeptin dynamics was more significant in patients who received dual reperfusion therapy (thrombolysis and thrombectomy) compared to those who received single reperfusion therapy (thrombolysis or thrombectomy) and those who received conservative treatment. Thus, the dynamic decrease in the level of copeptin at 24 h after the recanalization intervention may mirror the efficiency of the revascularization therapy. The main limitation of the study was the small sample of patients (34 patients) (44).

## 4 Discussion

Abnormalities in endocrine functions have been reported in stroke, activation of the hypothalamo-pituitary–adrenal axis being one of the first measurable physiological responses to cerebral ischemia (27). AVP is an indicator that reflects the activation of hypothalamic–pituitary–adrenal axis and it was shown to correlate with the state and prognosis of cerebral infarction (29).

Firstly, we assessed copeptin as a prognostic biomarker in acute ischemic stroke. The prospective studies that we analyzed on healthy patients who were followed-up for stroke occurrence showed *contradictory results*. On one hand, research has shown that men with diabetes and higher copeptin levels have a greater risk of developing stroke compared to those with lower copeptin levels (48). Furthermore, some studies have demonstrated an association between elevated copeptin levels and stroke risk, such as Fenske et al. (52), who observed a strong link in hemodialysis patients with type 2 diabetes. On the other hand, other studies did not find this connection; for example, Katan et al. (53) reported that markers like procalcitonin and MR-proANP were associated with a higher risk of stroke, but not copeptin. When evaluating for short-term outcome, long-term outcome (measured on mRS scale or with Barthel index) and mortality, most studies have shown that higher copeptin levels associate with: poor functionality (at discharge, at 3 months and at 1 year) and death. Other studies have shown a correlation between copeptin levels and ischemic lesion size, NIHSS score, age, stroke subtypes, personal previous pathologies, but the *results are inconsistent* (1, 6, 27, 37, 43, 54, 57, 60, 61). When evaluating for stroke recurrence, some predictive models were developed based on copeptin levels (4) and it was shown that copeptin may help for a better risk stratification for a recurrent cardiovascular event (23).

Secondly, we assessed copeptin in acute ischemic stroke patients depending on revascularization strategies. We found in the literature only one study that assessed the copeptin levels in consecutive moments of time in revascularized ischemic stroke patients (44). Their first conclusion was that copeptin level may mirror the efficiency of the revascularization therapy. Their second conclusion was that copeptin levels correlate with development of cerebral edema and hemorrhagic transformation. Both conclusions help in prognosis of revascularized ischemic stroke patients, but *the results should be replicated* on higher number of patients.

However, another limitation of dosing copeptin in ischemic stroke patients is that this biomarker can be significantly higher in many fluid disorders and stress-associated disorders (66).

Copeptin is secreted in equimolecular amounts with AVP and is considered a seric surrogate of AVP. Copeptin has the advantage of being easier to measure, stable *ex-vivo* and the result may be ready in approximately 1 h. Blek et al. (5) stated that measurement of copeptin is still not routinely performed in stroke patients despite many years of studies elucidating its association with stroke prognosis. This is due to the limitations given by previous studies: being carried out on small target groups and mostly in the eastern population.

The limitations of this review are primarily determined by the limitations of the included studies. The main limitations encountered in the literature were:

- Only few studies that took into account revascularization therapy (thrombolysis, endovascular treatment) and its relation to copeptin levels
- Only few studies took into account the time of onset of symptoms, the time until the patient presented to the emergency department, door-to-needle time, door-to neurologist time and their relation to copeptin levels
- Most studies were performed in rural areas and Eastern population
- Most studies were performed on stroke patients and further subgrouped depending on their imaging diagnosis (intracerebral hemorrhage, cerebral infarction, subarachnoid hemorrhage, others): further studies should focus on more targeted patients, with more strict inclusion criteria (such as: only ischemic stroke patients with cardioembolic mechanism)
- Only one study assessed stroke related complications such as brain edema and hemorrhagic transformation and their relation to copeptin levels: further studies should focus on finding “a troponin for brain” that could explain to physicians that a certain copeptin level at the moment of presentation may put the patient at risk for stroke-related complications, so they should target a more extensive therapy.
- There are only few studies that mention the use of vasopressin receptor blocker drugs that may reduce cerebral edema (41)

## 5 Conclusion

Prediction of long-term outcome at stroke onset only based by clinical deficits is difficult. This explains the need for useful blood biomarkers. Copeptin is a novel and promising biomarker for evaluating cerebrovascular diseases, even though multiple pathologies can lead to increased copeptin and it can be considered non-specific. However, if it is combined with other clinical or paraclinical parameters, the diagnostic accuracy may increase (67).

An early risk evaluation with an early estimation of the severity of stroke and prognostic of stroke is pivotal for individualized management, triage decisions, acute therapeutic management, optimized care and allocation of healthcare resources (27).

**Figure 2:**
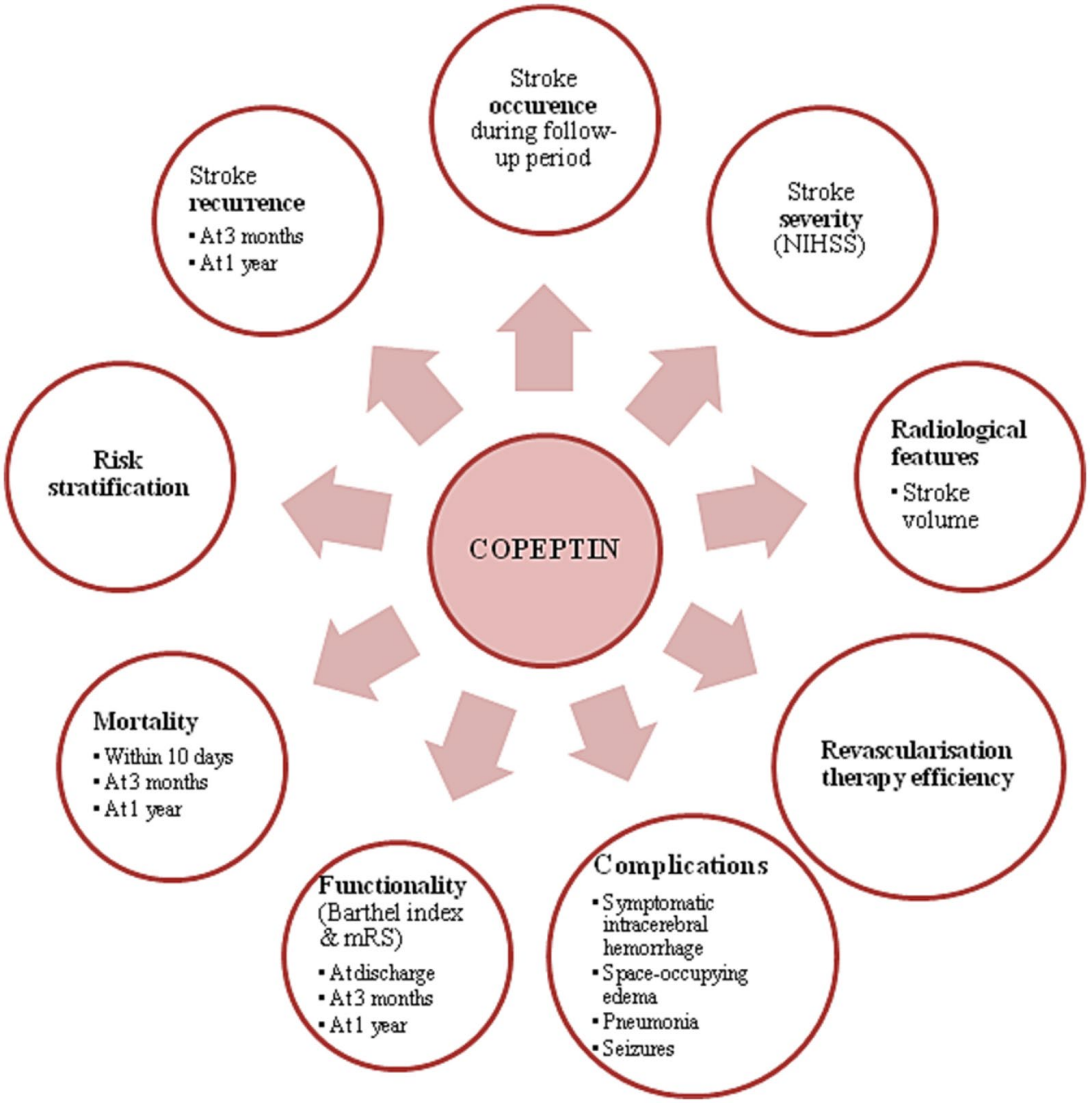
Benefits of copeptin use in ischemic stroke patients.

The above review explains that monitoring copeptin levels may help in several key moments for an ischemic stroke patient: prevention (copeptin predicting stroke occurrence among healthy individuals based on their comorbidities), moment of presentation (copeptin correlating with stroke severity and infarcted stroke volume), treatment efficacy, post-stroke complications, prognosis (short-term and long-term), mortality, risk stratification and stroke recurrence. A summary of the benefits of dosing copeptin in neurological practice are presented in Figure 2. In conclusion, copeptin may be a useful marker in ischemic stroke from a continuous point of view: *primary prevention* and *secondary prevention* (focusing on the moment of presentation in the *present*, but also focusing on the *future* of the patients).

## Data Availability

The original contributions presented in the study are included in the article/supplementary material, further inquiries can be directed to the corresponding author.
